# High-Fidelity Interactive Motorcycle Driving Simulator with Motion Platform Equipped with Tension Sensors

**DOI:** 10.3390/s25134237

**Published:** 2025-07-07

**Authors:** Josef Svoboda, Přemysl Toman, Petr Bouchner, Stanislav Novotný, Vojtěch Thums

**Affiliations:** Department of Vehicle Technology, Faculty of Transportation Sciences, Czech Technical University in Prague, Konviktská 20, 110 00 Prague, Czech Republic; tomanpre@fd.cvut.cz (P.T.); petr.bouchner@cvut.cz (P.B.); stanislav.novotny@cvut.cz (S.N.); thumsvoj@fd.cvut.cz (V.T.)

**Keywords:** motorcycle simulator, riding simulator experiment, motion platform, virtual reality, real motorcycle experiment, tension sensors

## Abstract

The paper presents the innovative approach to a high-fidelity motorcycle riding simulator based on VR (Virtual Reality)-visualization, equipped with a Gough-Stewart 6-DOF (Degrees of Freedom) motion platform. Such a solution integrates a real-time tension sensor system as a source for highly realistic motion cueing control as well as the servomotor integrated into the steering system. Tension forces are measured at four points on the mock-up chassis, allowing a comprehensive analysis of rider interaction during various maneuvers. The simulator is developed to simulate realistic riding scenarios with immersive motion and visual feedback, enhanced with the simulation of external influences—headwind. This paper presents results of a validation study—pilot experiments conducted to evaluate selected riding scenarios and validate the innovative simulator setup, focusing on force distribution and system responsiveness to support further research in motorcycle HMI (Human–Machine Interaction), rider behavior, and training.

## 1. Introduction

Interactive simulators currently represent an essential tool both in driver training and in research on Human–Machine Interaction, as well as in the development of advanced driver assistance systems (ADAS) [1,2]. In all these applications, the objective outputs from the simulator can be used to assess the performance of the tested subject or to objectively evaluate proposed solutions. Some of the most advanced simulator systems nowadays are utilized within the development facilities of world-class car manufacturers and their budgets reach up to tens of millions of EUR [3,4,5].

While driving simulators for cars or heavy machinery have become a standardized part of many applications, the development of realistic motorcycle simulators remains a significant technical challenge. As a single-track and inherently unstable vehicle, a motorcycle exhibits fundamentally different dynamic behavior compared to cars in the context of road transport. These specific characteristics impose demanding requirements on the mechanical design of the simulator, the complexity and accuracy of the virtual mathematical–physical model, and the types of feedback delivered to the rider [2].

An example of a hardware solution that enhances rider feedback is the use of a motion platform. Our previous study [6] has shown that the motion platform generally delivers a more realistic experience of virtual driving, which is essential for various types of experiments relying on objective measures of rider behavior. Additionally, virtual reality simulations using head-mounted displays (HMDs) offer a controlled yet immersive and realistic environment, enabling detailed behavioral analysis without exposing participants to real-world risks [7].

HMI and ARAS (advanced rider assistance systems) are becoming increasingly relevant in the context of motorcycle safety, training, and riding efficiency [8]. Unlike in cars, where such systems are already widespread, the integration of HMI and ARAS into motorcycles poses unique challenges due to the vehicle’s dynamic instability and reliance on constant rider input [9,10]. Advanced rider support must therefore be designed with precise sensitivity to timing and modality, considering how human behavior integrates with machine control in high-risk environments [11]. Recent investigations highlight the benefits of multimodal sensory feedback—particularly the coordination of visual and vestibular cues—which can enhance rider perception and reaction during complex maneuvers [12]. Simulator-based evaluations have further confirmed that immersive, behaviorally realistic feedback loops help riders interpret vehicle motion and improve performance [13]. In addition, empirical results underline that rider specific factors, such as posture and control strategy, significantly influence stability during slalom tasks, pointing to the necessity of personalization in ARAS design [14]. As these technologies evolve, HMI and ARAS solutions tailored for Power-Two-Wheeler (PTW) have the potential to become key instruments not only for safety enhancement, but also for adaptive training and rider development [8,12].

Historically, several motorcycle simulator concepts have been developed worldwide, providing valuable experience and inspiration for subsequent research. One of the earliest implementations was introduced by Honda in the late 1990s, presenting a motorcycle simulator mounted on a motion platform. The primary objective of this system was to offer a safe training environment for novice riders. A secondary, yet significant, aim was to facilitate research into the controllability and stability of single-track vehicles. To this end, a conceptual design incorporating four degrees of freedom was developed, and a prototype of a serial motion platform was constructed. This platform featured a total of seven movable axes, including the steering axis. To replicate longitudinal acceleration, a swing-based motion platform was proposed and realized using electromechanical actuators [15].

The advanced motorcycle simulator was also used as a tool for studying rider behavior at the University of Nottingham [16]. The hardware foundation of the simulator consists of a motion platform composed of two pairs of pneumatically driven cylinders arranged in parallel, which theoretically allow for tilts of up to 25 degrees. The simulator also incorporates a realistic motorcycle mock-up, including authentic control elements [17].

Among the most technologically sophisticated motorcycle simulators is the DESMORI system, developed by the WIVW institute. This simulator integrates a range of innovative technologies that provide comprehensive multisensory feedback to the rider. It was primarily designed as a research tool for evaluating rider workload during the development and testing of novel human–machine interface (HMI) technologies [2,18].

In this paper, we aim to present a new high-fidelity interactive motorcycle riding simulator, which integrates a motion platform, tension sensors, and immersive virtual reality via a head-mounted display (HMD), and to describe the methodology for its validation. The simulator has been evaluated through an experiment involving riders with varying levels of experience, with the aim of verifying its functionality in terms of technical accuracy, realism of interaction, and the relevance of the feedback provided mainly by the motion platform. Validation is carried out using three minimal scenarios representing fundamental types of riding situations. This work contributes to assessing the simulator’s applicability for research, training, and educational purposes, and provides a foundation for its further development and deployment.

## 2. Materials and Methods

### 2.1. Motorcycle Simulator Development

#### 2.1.1. Hardware Development

**Figure 1:**
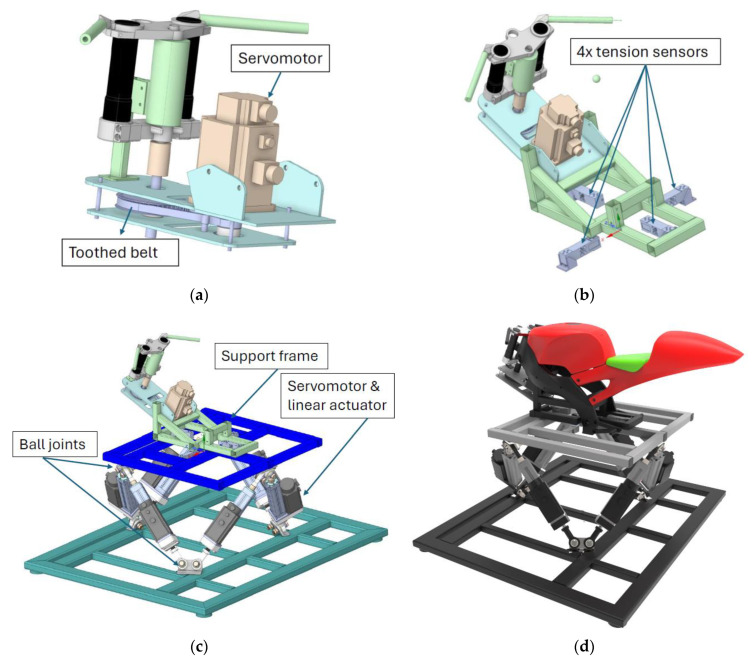
Final mechanical design of the simulator: (**a**) Steering transmission using a servomotor and a toothed belt at a pitch of 195 mm; (**b**) placement of tension sensors between the support frame and the upper part of the motion platform. The devices were arranged in a regular cross pattern with a spacing of 250 mm between them; (**c**) assembly of the motion platform and support frame; (**d**) final render of the complete simulator with the mock-up mounted.

The development of the simulator hardware involves the design and mechanical construction of the parallel 6DOF Gouth-Steward motion platform integrated with a motorcycle mock-up (based on Aprilia RS125, model year 2000, Aprilia S.p.A., Noale, Italy), as well as the implementation of individual sensors. The platform is equipped with tension sensors for real-time rider position monitoring, along with key control components implemented into mock-up including a steering servomotor, a throttle grip, front and rear brake sensors, a clutch, and auxiliary switches. The 3D CAD models of the mechanical construction of the simulator are shown in Figure 1.

Based on the mechanical configuration and tilt angle of the electromechanical actuators of the 6DOF motion platform, the following kinematic parameters were achieved—see Table 1.

As shown in Figure 1a, achieving an optimal steering torque required the implementation of a transmission system within the steering control. The transmission was realized using a toothed belt, with a total gear ratio of 1:2.4. This configuration enables a maximum achievable torque of 16.8 Nm at the handlebars, which is sufficient for realistic motorcycle steering simulation.

To enable more realistic steering behavior, strain-gauge-based rider position sensing was implemented into the mechanical structure. Strain gauges are utilized for control, as their output is integrated into the physical model of the simulator. The support frame is suspended from the upper frame of the motion platform using four tension sensors arranged in a cross configuration, with each sensor rated for a maximum load of up to 150 kg (see Figure 1b,c).

**Figure 2:**
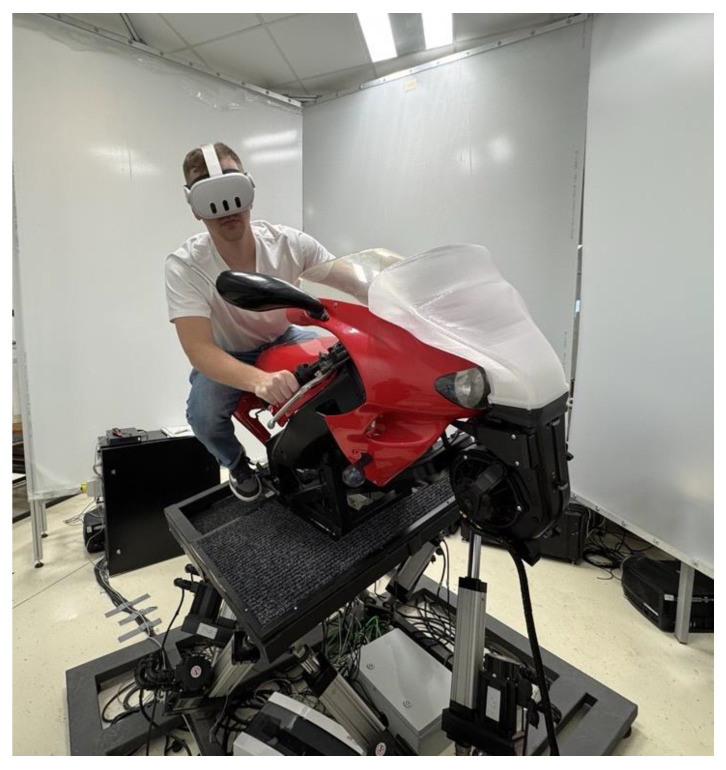
Complete hardware setup of the CTU MotoSim simulator (CTU in Prague, Faculty of Transportation Sciences, Czech Republic). Head wind system is placed in front of the rider.

The simulator design further incorporates a component for simulating external environmental effects—specifically, a high-performance fan system that communicates with the virtual environment via serial port. Based on the simulated riding speed, the fan generates airflow directed at the rider to simulate headwind see Figure 2.

The simulator visualization system is designed using a modular approach. The primary is a head-mounted display (HMD), for which we used devices such as the HTC Vive Pro Eye (HTC Corporation, Taoyuan, Taiwan) or Meta Quest 3 (Meta Platforms, California, USA), which provide sufficient resolution for immersive operation. In addition to HMDs, the simulator can also be operated using any large-format display panel or projection system.

#### 2.1.2. Software Development

**Figure 3:**
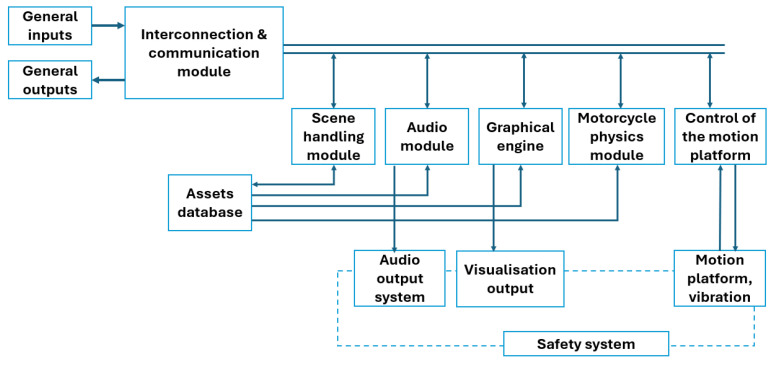
Modular software architecture of the CTU MotoSim motorcycle simulator [19].

The simulator software includes the development of a mathematical–physical model of the motorcycle and its integration with all simulator subsystems. The overall software architecture follows the systems developed by Driving Simulation Research Group at our faculty [19]. The architecture of the whole simulator system is defined by the structure presented in the block diagram in Figure 3.

**Figure 4:**
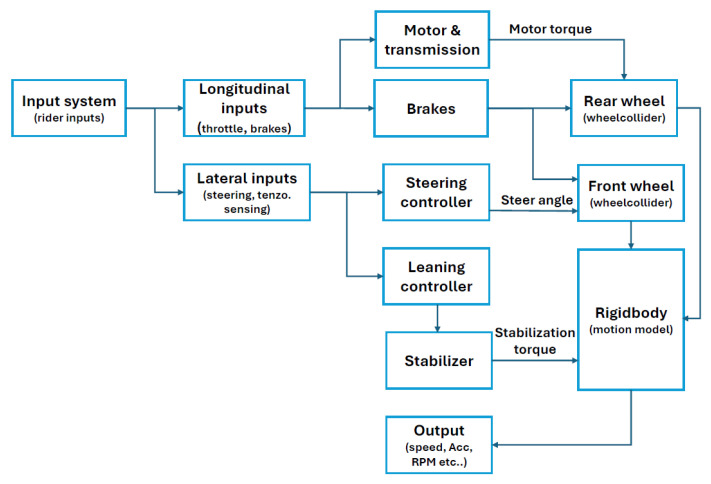
Block diagram of the virtual physical model of the motorcycle.

The simulation layer computes the virtual dynamics and renders the environment, while the control layer (PLC) handles real-time actuation and feedback. The development of the virtual environments as well as the mathematical–physical module was carried out within the Unity Engine (v2022.3.18), using C# scripting. The physical module of the virtual motorcycle consists of several core components. The block diagram (Figure 4) illustrates the virtual physical model of a motorcycle and the data flow between its individual modules. In the input layer, the control system receives rider-generated signals, which are classified based on their influence on vehicle dynamics, either longitudinal or lateral. Longitudinal inputs, such as throttle, braking, and clutch control, are essential for producing torque on the wheel models (WheelColliders), with the sign of the torque (positive or negative) determined by the direction of wheel rotation. Lateral inputs include handlebar steering and signals from strain-gauge sensors. These are processed first by the Steering Controller and subsequently by the Leaning Controller, which, together with the Stabilizer module, manages the lean dynamics of the virtual rider–motorcycle system. The resulting lean torques are applied to the rigid body, which represents the central component of the physical model of the motorcycle, that is, the vehicle body with defined mass properties, including total mass and center of gravity (CoG). The model outputs key physical parameters such as speed, acceleration, RPM of the engine, etc., which serve as inputs for visualization, feedback loops, and motion platform actuation.

For visual output, a camera component is used, and for audio rendering, an Audio Listener is included. All components are parameterized and controlled via custom-developed C# scripts developed specifically for the purposes of this simulator. The calibration of the longitudinal dynamics of the physical model is described in detail in our previous work [20,21]. Simulation time is fixed-step, synchronized with the physics update cycle in Unity, and decoupled from visual frame rate to prevent motion inconsistencies. The virtual environment of the simulator was created using a combination of proprietary and third-party assets—see Figure 4. To ensure smooth rendering performance, all scene objects were optimized to maintain a frame rate of no less than 70 FPS (In the case of using an HMD with a resolution of 2064 × 2208 pixels per eye). Special attention was given to objects positioned near the track, as these play a key role in enhancing the rider’s perception of speed. This perceptual effect is further supported by the audio system, which combines engine sounds with aerodynamic noise and additional elements such as braking sounds. In addition, special effects were integrated into the virtual scene to enhance realism. These include, for example, rider hand animations that respond to throttle input, braking, and similar control actions.

#### 2.1.3. Simulation Behavior, Input Handling, and Motion Control

The CTU MotoSim simulator allows directional control through both handlebar steering, primarily at lower speeds, and the intentional lean of the rider into the corner, a technique commonly referred to as body steering. To ensure that the steering feedback on the handlebars authentically reflects real riding conditions, the stiffness of the steering servomotor is continuously modulated by the control PLC as a function of the motorcycle’s speed and current lean angle. The strain gauges are arranged in a cross configuration within the mechanical structure, allowing rider position sensing along both the lateral and longitudinal axes. Before each ride, the rider is “weighed” in the neutral position to calibrate the strain gauges and establish a zero-reference point. The output of the strain-based sensing system is a vector characterized by its magnitude and angle. Using these parameters, the rider’s position in both axes can be computed through trigonometric functions.

**Figure 5:**
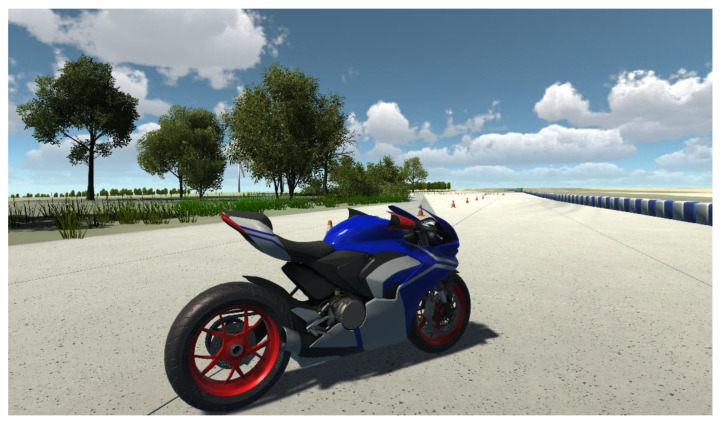
Virtual scene incorporating custom-made and third-party assets.

As illustrated in the system architecture diagram (Figure 5), communication between individual modules is managed by a communication module, which handles most of the system’s inputs and outputs. It interfaces with the other modules of the simulator using communication protocols such as UDP, HTTP requests, and serial port communication. An example of such communication is transmitting outputs from the mathematical–physical model via the UDP protocol to the control system which controls the motion platform, tension sensing, and steering servo system. This data packet contains information on the position, velocity, and acceleration of the motorcycle model across all axes of translational and rotational motion and is transmitted at a sampling frequency of 125 Hz.

The motion control system is based on a fully autonomous industrial PLC running dedicated control software. The motion platform actuators’ controllers are pulse-driven via the PLC, which executes the motion control algorithm, signal filtering routines, and a washout algorithm. The motion platform control algorithm was developed and described in publication [22]. For the purposes of the motorcycle simulator, the constants of the washout algorithm, as well as some other variables, were tuned to achieve the fastest and most realistic system response possible. As acceleration signals are derived from time differentiation of velocity, they are inherently noisy and thus require appropriate filtering. To smooth the acceleration data, a simple low-pass filter is applied in the following form:

y(t) = y(t − 1) + ω + (u(t) − y(t − 1))·Δt,(1)

where u(t) is the current input value, y(t) is the filtered output, Δt is the sampling time step, and ω is the weighting coefficient defined as ω = 2πf, with f being the cutoff frequency that is typically set between 2 and 4 Hz. This filter acts as a first-order smoothing function. This filter introduces only minimal phase delay, which makes it suitable for real-time control of the motion platform.

When the motorcycle simulator is operated with both an HMD and a motion platform, it is necessary to compensate for the position and rotation of the virtual camera relative to the virtual motorcycle model. Without these compensations, the rider’s perceived position drifts with respect to the virtual motorcycle, which degrades the overall realism of the simulation. A separate aspect is the rider’s leaning, which is conveyed both through the physical tilting of the motion platform and by tilting the horizon within the virtual environment, typically at a greater angle to enhance the perceptual effect.

### 2.2. Experiment

#### 2.2.1. Experiment in Virtual Environment Using the Motorcycle Simulator

**Figure 6:**
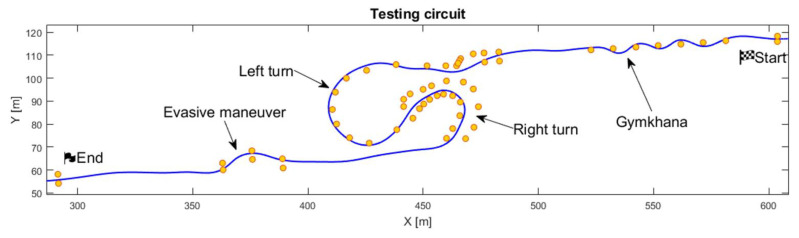
The test track was used for the experiment, comprising four sections with minimal riding scenarios: Gymkhana, long sweeping left/right turn, and evasive maneuver. Yellow markers represent traffic cones that outline the track. The blue line indicates the trajectory of the rider.

The experiment conducted in virtual reality using the motorcycle simulator was performed on a virtual test area, with the track itself delineated by traffic cones. The layout was divided into three main sections, each representing a fundamental riding scenario. The validation methodology of motorcycle simulators using minimal scenarios, as proposed by Will et al. [18], is intended to allow a comprehensive (global) assessment of the capabilities of the simulator and guide its subsequent optimization. In our study, we adopted this approach by selecting three minimal scenarios: the Gymkhana section, the constant-speed cornering section, and the evasive maneuver section; see Figure 6.

The experiment was conducted with a total of 13 participants, representing a wide range of riding experience—from less experienced riders to highly skilled individuals, including competitive racers. Prior to the experimental rides, all participants underwent standardized training to familiarize themselves with the simulator and the task structure. The training phase lasted approximately 15–20 min per participant. After training, each participant subsequently completed four measured runs—two with the motion platform activated and two with the platform deactivated, i.e., using the simulator in its static mode. The order of simulator tests with and without platform was equally randomized across subjects. After completing the test rides, all participants were presented with a questionnaire designed to subjectively assess different dimensions of the simulator experience. The questionnaire is included in Appendix A. The order of the motion and static simulator runs was counterbalanced across participants to mitigate potential order effects.

During the measurement rides, two main data sources were recorded: driving data from the simulation and data from external sensors. The first external device was the Xsens MTi-680G (Xsens Technologies B.V., Enschede, The Netherlands) measurement unit [23]. The second was the Xsens MTw Awinda (Xsens Technologies B.V., Enschede, The Netherlands) [24], which was attached to the HMD and used to track the rider’s head movement. An overview of all recorded variables is provided in Table 2. All data were logged at a consistent sampling frequency of 100 Hz.

#### 2.2.2. Test Track Experiment

The experiment was carried out under real-world conditions at the Panensky Tynec airfield. The test track was constructed to match the layout used in the virtual reality experiment; see Figure 5. A total of 10 participants were involved in the study, consisting primarily of experienced motorcycle riders. Riders were provided with enough time for training and adaptation to the track layout and motorcycle control before the start of the measured runs. Subsequently, each rider completed two measured runs. The experiment was conducted in summer at a temperature of 32 degrees Celsius and 18% humidity.

**Figure 7:**
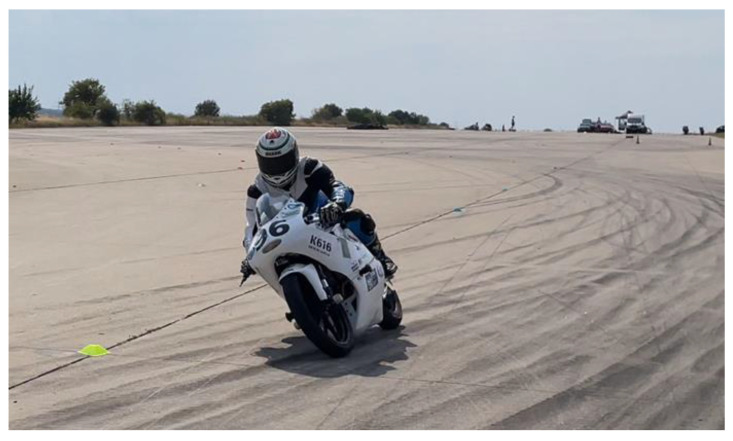
The test was performed with the CTU Lions Evo 2 Electric motorcycle (CTU in Prague, Faculty of Transportation Sciences, Czech Republic) on a track defined by traffic cones, replicating the same layout as in the simulator-based experiment [25].

The motorcycle used for the real-world experiment was the CTU Lions Electric Evo 2 racing prototype [25], whose geometric and mass properties closely resemble those of the simulator mock-up (Figure 7).

During the real-world experiment, the recorded data closely matched that of the simulator-based study. The Xsens MTi-680G measurement unit was also used in this case. The position of the motorcycle along the track was determined using GNSS data (longitude, latitude, altitude). Furthermore, the measurements included acceleration on all three axes (X, Y, Z), local rotations (roll, pitch, yaw), motorcycle speed, throttle position, and brakes input.

## 3. Data Interpretation Analysis and Discussion

In this chapter data interpretation analysis and discussion are presented based on objective and subjective acquired data.

### 3.1. Objective Data Assessment

**Figure 8:**
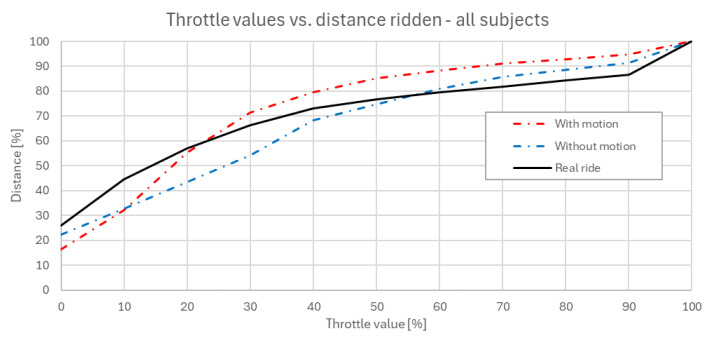
Normalized number of trajectory segments ridden with respective throttle values used by riders [%].

Figure 8 shows how much throttle (value in %) corresponds to the number of segments ridden by riders. Due to different lengths and numbers of segments of each rider, this number was normalized and expressed in percents. The curve is ascensive due to its cumulative sum character. Data suggests that the behavior of riders when controlling the throttle in the simulator does not differ significantly from real-life riding. The differences between riding with and without the motion platform do not manifest as a consistent change in average performance or trend, but rather in the variability of control.

This trend suggests that riders were more conservative in their throttle application when receiving full motion feedback, likely due to an increased perception of both accelerations, longitudinal and lateral. The combined effect of visual and inertial cues appears to enhance the realism of the ride, causing participants to modulate throttle input more carefully. In general, riding with an active motion platform can be characterized as a deliberate in the way of throttle opening application.

**Figure 9:**
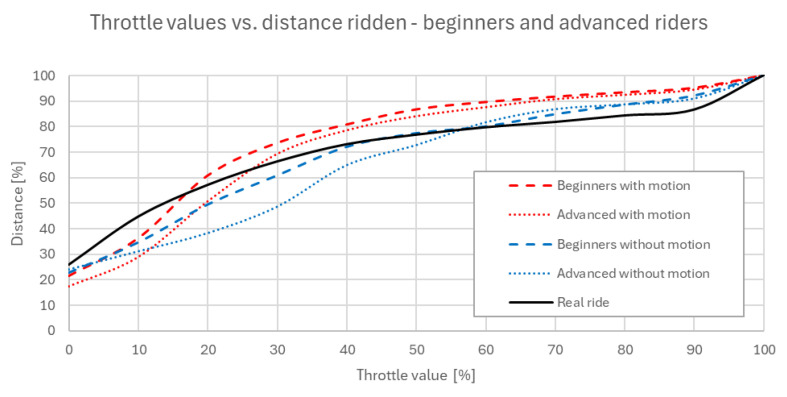
Normalized number of trajectory segments ridden with respective throttle values used by riders [%]—comparison of beginner and advanced riders.

The following Figure 9 provides a more detailed examination of the curves presented in Figure 7, but separated by rider experience level: beginners (up to 5 years of active practice) and advanced riders (more than 5 years of active practice). The motion platform appears to bring results of both groups of riders closer together in terms of their throttle control behavior.

To sum up riders’ throttle control behavior in the experiment, higher throttle openings, which occur over shorter sections of the ridden distance, show a closer resemblance between the real ride and the simulator ride with active motion platform. On the other hand, lower throttle values, which appear over a larger portion of the ridden trajectory, result in the real ride more closely aligning with the simulator ride without the active motion platform. This indicates that in less dynamic phases, the absence of motion cues has a smaller impact on rider behavior. Overall, the real ride pattern lies in between the two simulated conditions, sharing characteristics with both, but leaning toward the motion-enabled simulation in high-intensity sections and the passive one in steadier phases. This suggests that the simulator in both configurations provides a close approximation of real riding behavior.

The following Table 3 summarizes the results of the simulator-based experiment, expressed as mean values across all participants. The table is structured according to three minimal riding scenarios and further segmented by the motion platform condition—either active or inactive. Each participant performed two measured rides with and without the motion platform activated. To evaluate the significance of the effect of motion and consistency of data, a paired *t*-test was performed. The paired sample *t*-tests within each condition (with and without motion) generally showed no statistically significant differences between the first and second ride, suggesting that participants adapted to both setups. The results indicate that in the absence of motion feedback, riders tended to perform sharper maneuvers. This observation is supported by higher values of resultant acceleration in the static scenario. Additionally, higher average, entry, and exit speeds were observed when the motion platform was disabled. Also, average speed and exit speed are consistently affected by the presence of motion, with significant *p*-values in all sectors. Additionally, average total acceleration also shows strong significance in Sectors 1 and 3 (*p* = 0.003 and *p* = 0.004), confirming that motion feedback leads to smoother, less aggressive riding. These findings suggest that riders may not fully perceive the dynamic behavior of the motorcycle without physical motion cues, even when immersed in a visually comprehensive virtual environment delivered via an HMD.

The table also includes lean angle values for each maneuver, as presented to the rider through the HMD. These lean angles were intentionally reduced within horizon tilts associated with higher lean angles. These angles were found to increase the symptoms of motion sickness in experiment participants. This adjustment was implemented to ensure greater user comfort while maintaining sufficient visual cues for cornering perception.

**Figure 10:**
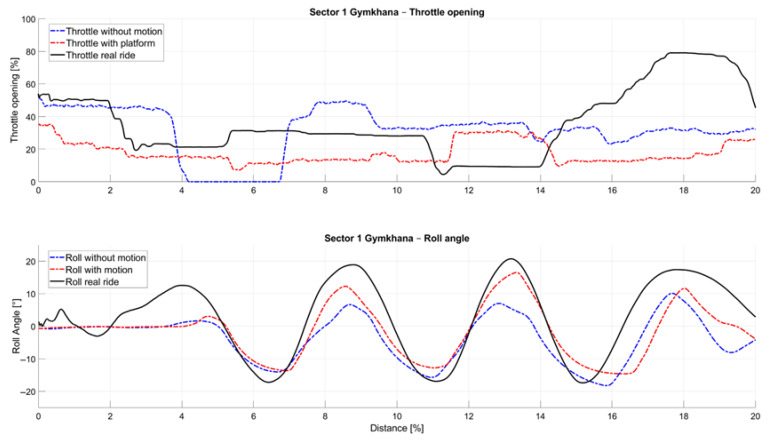
Sector 1 Gymkhana—Detailed analysis of throttle opening and rolling angle of selected advanced rider.

The following Figure 10 presents a detailed look into the Sector 1 Gymkhana of the selected advanced rider and brings the comparison of both simulated and real polygon rides. In the scenario without an active motion platform, throttle control exhibits greater uncertainty, reflected in a less precise and more variable throttle opening pattern. This is indicative of a diminished perception of acceleration forces, which likely contributes to inconsistent rider input. Conversely, when the motion platform is active, throttle modulation tends to be overly cautious, suggesting that the enhanced vestibular feedback leads riders to adopt a more conservative approach to throttle opening. This effect is particularly visible during initial acceleration phases, where the simulation without the active motion platform reveals a tendency toward higher throttle openings followed by abrupt closures.

A detailed look at the roll angle in the Figure 10 data reveals that the counter-steering effect, clearly present at the beginning of the section in the real-ride measurements, is difficult to reproduce in both simulator conditions and is absent in the simulated rides. Furthermore, when examining the roll angles themselves, a noticeable offset is observed: the lowest lean values occur in the simulation without the active motion platform, while higher lean angles are recorded during rides with the active motion platform that are closer to angles measured in real rides. These findings suggest that the presence of the motion platform enhances the fidelity of lateral dynamics representation.

**Figure 11:**
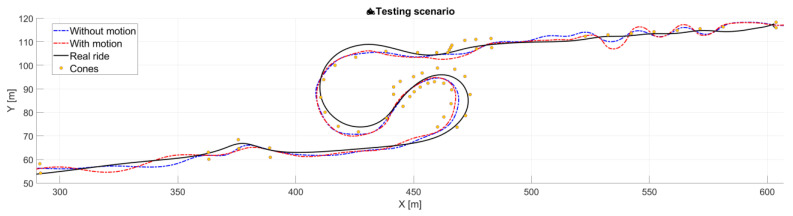
The test track—Illustration of trajectories of selected advanced rider in both simulated rides and real polygon ride. The real ride trajectory is burdened with inaccuracies arising from GNSS signal limitations during the recording.

An illustration of the overall trajectories of a selected advanced rider is presented in Figure 11. The real-ride trajectory is burdened with inaccuracies arising from GNSS signal limitations during the recording. Despite this, the overall shape of the trajectories is mutually consistent, indicating that the simulated rides—both with and without motion platform activation—reflect the dynamics of real-world motorcycle operation with sufficient fidelity.

The following Table 4 shows a comparison of the times achieved by a group of advanced riders in individual track sectors during both examined, simulated, and real riding conditions: without an active motion platform, with an active motion platform, and during a real ride.

In Sector 1, riders completed the sector faster without the motion platform, while the times with the motion platform and during the real ride were very similar, but with higher variance. This indicates that the motion platform simulation approximates the real riding experience in this segment more closely than the non-motion condition. In Sector 2, a similar trend is observed where riding using the simulator with motion is closer to the real ride. It is also repeated that data from the active motion platform scenario and real ride show higher data variance. In Sector 3, real rides took significantly longer than either simulation, with the motion platform condition showing slightly longer times than the non-motion condition. In this sector, riders tended to accelerate much stronger at the end of the evasive maneuver, which was most likely caused by nearing the end of the tested scenario.

### 3.2. Subjective Data Assessment

The next part of data interpretation analysis and discussion focuses on subjective data gathered during the simulator riding experiment. Data for the presented assessments are based on the Experiment Questionnaire in Appendix A.

**Figure 12:**
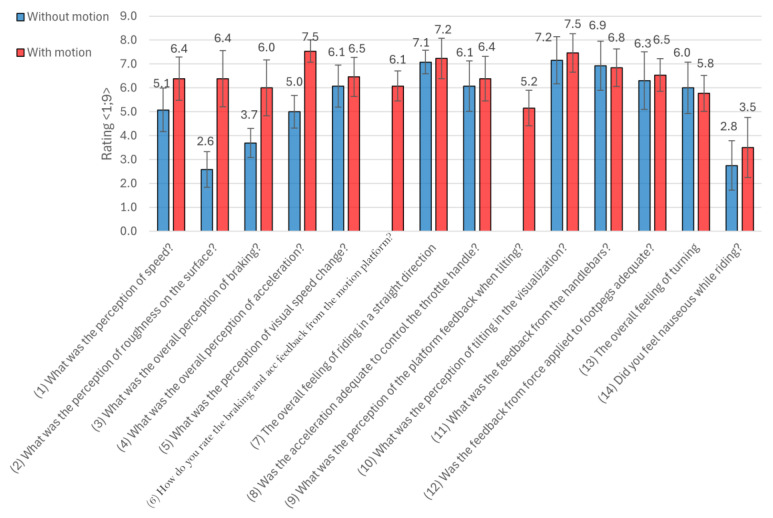
Subjective evaluation of simulator rides with active and non-active motion platforms.

Overall comparison of the tested scenarios evaluates questions from 1 to 14 in Figure 12. Questions 6 and 9 are targeted directly to motion platform participants, thus there are results presented only for the simulator ride with the active motion platform. Subjects of the questionnaire responded on scale from 1 to 9. Results are based on 13 subjects with a mean age of 31.77 years and mean value of riding practice 8.50 years. Furthermore, subjects are divided into two groups of riders, up to 5 years of practice and more than 5 years of practice, for detailed evaluation.

Figure 12 shows the evaluation of the longitudinal and lateral dynamic perceptions of all subjects. Subjective questionnaire ratings show that the active motion platform significantly influences the perception of key aspects of the simulated ride in terms of speed perception, surface roughness, braking, and acceleration. This suggests that the motion platform significantly contributes to physical feedback during changes in speed and longitudinal vehicle dynamics. In terms of lateral dynamics evaluation, we observe similar results for both analyzed simulator rides. Focusing on the evaluation of the motion platform effects when leaning (Question 9), we can compare it with the result of motion platform feedback while braking and accelerating (Question 6). From this point of view, we observe a slightly lower value by 0.9. Overall, similar results were obtained for questions of visualization, handlebars, footpeg effects, and feedback across both types of rides.

The following Figure 12 summarizes the subjective ratings of three types of minimal scenarios (Gymkhana, left/right turns, and evasive maneuvers)—with less than 5 years of practice and with more than 5 years of practice—and compares the results when riding without and with the active motion platform. Across all maneuver types, a consistent trend can be observed: activating the motion platform tends to increase the perceived difficulty of the task. For example, in the Gymkhana maneuver, the average subjective difficulty rises from 4.4 to 5.6 when motion is enabled. Similar increases are observed in left/right turns (from 4.8 to 5.7) and evasive maneuvers (from 4.5 to 5.5). These results suggest that the additional input provided by the motion system may challenge the rider’s ability to maintain control, particularly in more complex maneuvers. The perception of control tends to decrease slightly with the motion platform across tasks, with the most notable reduction occurring in Gymkhana (from 6.6 to 5.7). Ratings imply that although the motion system enhances realism, it may also introduce a higher load that affects riders’ confidence and execution. Similarly, perceived riding smoothness consistently declines in all maneuver types when motion is active. The drop is most evident in Gymkhana (from 6.1 to 5.1) and evasive maneuvers (6.4 to 5.6), likely due to the increased demand for the precise coordination of movements in maneuvers. In contrast, the change is less pronounced in turning (5.7 to 5.5), indicating that platform-induced motion may have a less disruptive effect in this narrower task.

It is important to note that several participants reported greater ease of control when riding without the motion activated platform. However, this came at the cost of perceived realism—riders frequently described the motionless simulation as lacking in authenticity, particularly during dynamic maneuvers. Furthermore, a small number of subjects experienced mild symptoms of discomfort or nausea during rides with the active motion platform.

**Figure 13:**
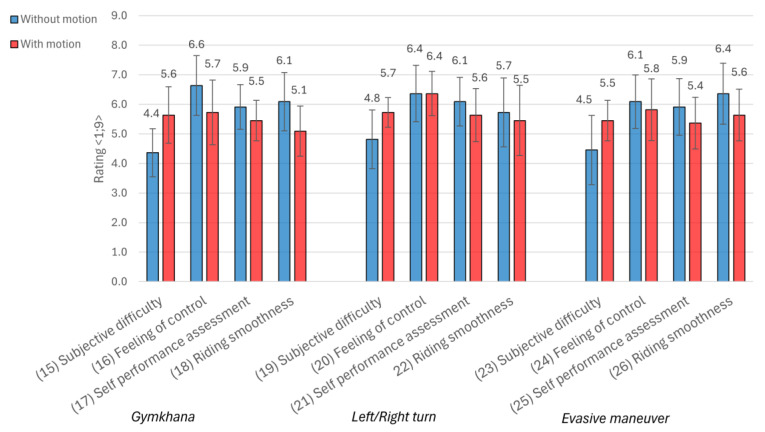
Subjective evaluation of simulator rides with active and non-active motion platform in 3 examined sectors—Gymkhana, Left/Right turn, Evasive maneuver.

Comparing subjective data from Figure 13 with results from Table 3, riders clearly perceived the motion ride as more difficult, less smooth, and with less control, which corresponds to the decrease in speed and average and max accelerations in data in the Gymkhana sector. A similar effect was observed in the second and third sector. Although the feeling of control was almost the same, riders reported higher difficulty and lower smoothness, which again correlates with a decrease in speed and average and maximal accelerations. The motion platform therefore enhances the realism of the simulation but also imposes greater demands on the rider—demands that are reflected both in their perceived experience and in their measurable performance.

**Figure 14:**
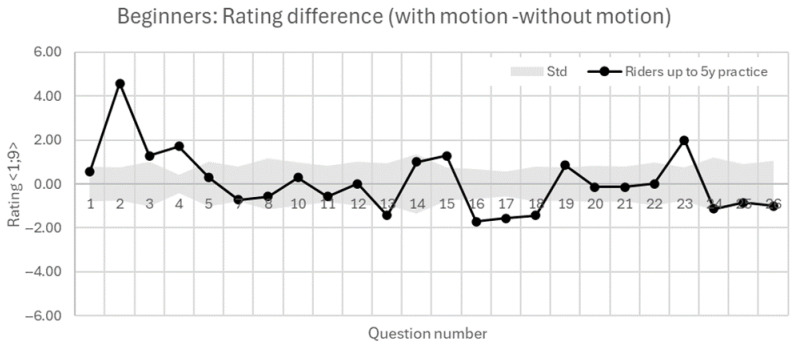
Subjective evaluation of beginner group of riders with less than 5 years practice. Results show the difference in rating in the scenario with motion against the without-motion ride.

**Figure 15:**
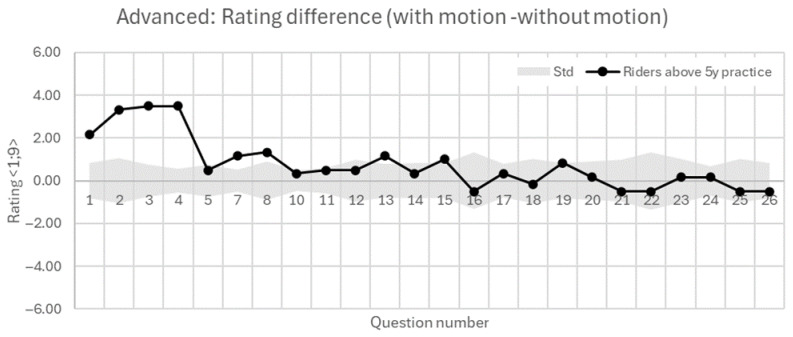
Subjective evaluation of group of Advanced riders with practice above 5 years. Results show the difference in rating in simulator ride with motion against the without motion ride.

The following Figure 14 and Figure 15 show the results of beginners and advanced riders based on their active motorcycle riding practice in years. These figures show the mutual difference in the evaluations of riding with the motion platform activated minus riding without the motion platform. In terms of perceptions of speed, braking, and acceleration, advanced riders (>5 years) show substantially greater increases in perceived speed (+2.17), braking (+3.5), and acceleration (+3.5) due to motion, compared to less experienced riders (<5 years), who show more moderate increases (+0.57, +1.29, and +1.71, respectively). This suggests that experienced riders are more sensitive to motion cues, potentially due to their stronger internal models of real riding dynamics.

Advanced riders report improved feelings of straight-line riding and throttle adequacy with motion (+1.17, +1.33), while beginners show small or negative changes (−0.71, −0.57), possibly indicating initial confusion or difficulty adapting to motion feedback. Beginners report a negative change in handlebar feedback (−0.57), whereas experienced riders report a slight improvement (+0.5). Footpeg feedback differences are minimal across both groups. Furthermore, beginners report a drop in the overall feeling of turning with motion (−1.43), whereas experienced riders improve (+1.17). Visual leaning perception increases only marginally in both groups. It is important to mention that these evaluations differences are, in general, very small, and are at the boundary of the standard deviation interval.

Focusing on minimal scenario specifics, we observe very small mutual differences at the boundary of the standard deviation interval as well. Both groups show small increases in perceived difficulty and minimal change in control and smoothness while evaluating left and right turning. In the case of Gymkhana, it is worth mentioning that the feeling of control drops significantly for beginners (−1.71), but only slightly for advanced riders (−0.5). The highest differences occur in evasive maneuver. Beginners show increased difficulty (+2) and declines in performance and smoothness (−0.86, −1.0), while experienced riders show only minor effects.

All in all, to compare both groups, advanced riders (>5 years) appear to benefit more from motion-platform activation, reporting slightly higher realism, control, and improved perception in key domains like speed, braking, and cornering. Less experienced riders (≤5 years) on the other hand, while noticing motion effects more, tend to struggle with control and performance perception in more complex tasks. Their feedback suggests possible cognitive overload or mismatch with expectations, especially in technical exercises. Both subjective evaluations from Figure 14 and Figure 15 and objective data from Figure 9 show that the active motion platform increases the immersivity of the simulated ride, which is reflected in more careful throttle opening. Beginners are more sensitive to physical feedback in this regard. Moreover, in the case of beginners in the static mode, it was observed that their perception of speed—both in absolute terms and in terms of visual speed variation—was generally lower compared to advanced riders. This was accompanied by a subjectively reported higher perception of deceleration, which may have resulted in more frequent or pronounced throttle overshooting, potentially linked to an increased confidence in the braking effect.

Generally, motorcycle riding is inherently demanding, and mastering the simulator requires a dedicated learning process. Initially, novice riders tend to simplify their control strategies to manage the task more easily. Experienced riders, on the other hand, strive to replicate real motorcycle riding techniques as closely as possible within the simulator environment. However, this does not guarantee that over time they will maintain a realistic riding style; without sufficient challenge or realistic feedback, even skilled riders may gradually adopt a more “arcade-like” style of control. The desire to ride exactly as on their own motorcycle often conflicts with the limitations of the simulator, and as a result, riders adapt their behavior towards a simplified, less realistic approach to control. This adaptation process highlights the importance of maintaining high-fidelity feedback and appropriate tasks to preserve realistic riding habits during simulator training.

## 4. Conclusions

This study presents the development and experimental validation of the CTU MotoSim motorcycle simulator, a high-fidelity system combining a six-degrees-of-freedom motion platform with a physically accurate motorcycle mock-up and immersive virtual environment. The hardware integrates rider-interactive components such as a steering servomotor, throttle grip, brake and tension-based rider position sensing, and head-mounted display visualization. The software architecture employs a modular approach, incorporating a physics-based model developed in Unity and controlled via an industrial PLC that governs motion feedback, input handling, and real-time signal processing.

To evaluate simulator realism and rider interaction, two experiments were conducted: one in a virtual environment using MotoSim, and a comparative test on a real-world track using the CTU Lions Electric Evo 3 motorcycle. Both experiments employed matched track layouts and a standardized data acquisition setup, including Xsens inertial sensors and rider input logging. Participants with varying skill levels completed a series of controlled scenarios designed to reflect essential riding tasks, such as Gymkhana, constant-speed cornering, and evasive maneuvers. The results form a baseline for assessing simulator performance and fidelity and provide a foundation for further HMI research and rider behavior studies in controlled yet realistic training environments. This study focuses on the initial validation of hardware input peripherals, with particular emphasis on the strain-gauge sensing system. Accordingly, a set of minimal experimental scenarios was employed to reflect this early validation stage. Future research will aim to incorporate more complex and dynamic conditions to further evaluate system performance and rider interaction. From the point of view of the rider, the simulator provides strain-gauge data that are planned for future development of the simulator in the way of more advanced control algorithms and the enhanced integration of sensor data into the physical model.

This study investigated the impact of motion feedback on rider behavior, control performance, and perception within the development of a new high-fidelity motorcycle simulator, using both objective data and subjective responses. The analysis considered differences between simulated rides with and without an active motion platform, real-world riding conditions, and the experience level of the riders. Objective data analysis revealed that throttle control behavior in the simulator generally aligns with real-world patterns. Riders exhibited more careful and modulated throttle opening control when motion cues were present, likely due to the enhanced perception of primarily longitudinal and lateral accelerations. Interestingly, throttle control behavior among beginners and experienced riders showed convergence when motion was enabled, regardless of prior experience. Sector-based analysis further confirmed that simulator rides with motion generally approximate real ride behavior more closely than those without motion, particularly in high-intensity sections. However, in some cases, riders performed quicker maneuvers in the absence of motion feedback, indicating that the lack of physical cues may encourage overconfident or exaggerated control actions. Lean angle analysis demonstrated that simulated rides without motion tended to produce shallower lean values. Nevertheless, some dynamic elements, such as the initiation of counter-steering, remained challenging to reproduce accurately in the simulator. This emphasizes the complexity of replicating nuanced riding dynamics even in high-fidelity virtual environments.

Subjective evaluations further reinforced these findings. Riders consistently reported greater perceptions of speed, braking, and acceleration when the motion platform was active. While this enhanced realism, it also increased perceived task difficulty. Importantly, experience level played a role in shaping subjective responses. Advanced riders (>5 years of practice) showed higher sensitivity to motion cues, reporting stronger perceptions of acceleration, braking, and speed. On the other hand, less experienced riders (≤5 years) reported declines in control confidence and perceived smoothness when motion was active. These differences suggest that while motion feedback appears in the simulation experience, it may also overwhelm beginners with physical riding dynamics, leading to a less stable performance.

This research demonstrates that integrating motion feedback into motorcycle simulations meaningfully enhances the fidelity of the riding experience, particularly for experienced users. To maintain a realistic riding style and avoid a shift toward simplified or “arcade-like” control patterns over time, simulator environments must provide continuous challenges, accurate physical feedback, and contextually appropriate task difficulty. In conclusion, high-fidelity simulators equipped with active motion platforms hold significant promises for rider training and research, with careful attention to both technical fidelity and user adaptation.

## Figures and Tables

**Table 1:** Kinematic properties of the designed motion platform.

	Position	Velocity	Acceleration
Pitch	±16°	±38°/s	±584°/s^2^
Roll	±17°	±41°/s	±626°/s^2^
Yaw	±24°	±61°/s	±940°/s^2^
Surge	±177 mm	±550 mm/s	±8.5 m/s^2^
Sway	±195 mm	±490 mm/s	±7.5 m/s^2^
Heave	±123 mm	±320 mm/s	±4.9 m/s^2^

**Table 2:** Overview of objective data recorded during the experiment.

Measured Quantities	Variable	Units
Motorcycle position	Coordinates (X, Y, Z)	[m]
Motorcycle local rotation	Euler angles (roll, pitch, yaw)	[o]
Motorcycle velocity	Speed	[m·s^−1^]
Motorcycle acceleration	AccX, AccY, AccZ	[m·s^−2^]
Primary rider inputs	Steering angle	[o]
	Throttle input	[%]
	Brakes input	[%]
Rider position data	Strain gauges angle	[o]
	Strain gauges vector magnitude	[/]
	HMD roll	[o]
External sensor Xsens MTI-680	Euler angles (roll, pitch, yaw)	[o]
	Motion platform accelerations (X, Y, Z)	[m·s^−2^]
External sensor Xsens Awinda	Head rotations (Roll, Pitch, Yaw)	[m]
	Head accelerations (X, Y, Z)	[m·s^−2^]

**Table 3:** Data measured within the simulator experiment. Average speed values are presented for each sector. “Entry speed” and “Exit speed” indicate the velocities at the entrance and exit of each riding section. “AvgAccTotal” and “MaxAccTotal” represent the average and maximum values of the total acceleration magnitude, calculated as the resultant of longitudinal and lateral accelerations. In the sections without and with motion, the *p* values represent results of paired sample *t*-tests across two rides. The comparison column presents results of paired sample *t*-tests for the effect of motion.

Variables	Without Motion			With Motion			Comparison
	Mean	SD	*p* Value	Mean	SD	*p* Value	*p* Value
Sector 1: Gymkhana							
Avg speed	28.54	3.10	0.948	23.67	3.97	0.767	**0.003**
Exit speed	40.07	6.88	0.518	34.19	8.31	0.145	**0.015**
Entry speed	13.99	5.15	0.457	13.52	3.66	0.766	0.096
Max roll	10.02	4.34	0.673	11.53	3.55	0.292	**0.091**
AvgAccTotal	1.76	0.38	0.432	1.27	0.43	0.501	**0.003**
MaxAccTotal	4.11	1.73	0.697	2.92	0.76	0.774	0.195
Sector 2: Left/Right turn							
Avg speed	42.91	6.62	0.113	37.38	8.55	0.455	**0.001**
Exit speed	54.19	12.21	0.828	46.44	11.83	0.655	**0.009**
Entry speed	44.99	8.30	0.189	37.66	9.84	0.098	**0.012**
Max roll	14.07	6.35	0.124	14.07	4.54	0.941	**0.012**
AvgAccTotal	1.37	0.32	0.131	1.08	0.41	0.171	**0.001**
MaxAccTotal	6.68	3.77	0.350	3.39	1.57	0.206	**0.026**
Sector 3: Evasive maneuver							
Avg speed	48.55	12.29	0.655	43.36	8.57	0.383	**0.043**
Exit speed	52.99	7.76	0.725	47.85	7.36	0.101	**0.021**
Entry speed	53.21	13.39	0.838	46.39	10.88	0.897	**0.027**
Max roll	11.92	7.22	0.923	13.62	7.90	0.083	0.272
AvgAccTotal	2.28	0.76	0.319	1.89	0.63	0.528	**0.004**
MaxAccTotal	4.40	2.61	0.829	3.71	1.94	0.917	0.196

*p* Values < 0.05 are reported in boldface.

**Table 4:** Sector times comparison of group of advanced riders.

	Without Motion	With Motion	Real Ride
Sector 1	11.97 s (SD 0.69)	13.53 s (SD 1.63)	13.47 s (SD 1.51)
Sector 2	21.94 s (SD 0.84)	22.88 s (SD 2.71)	24.14 s (SD 3.07)
Sector 3	6.56 s (SD 0.48)	6.95 s (SD 0.41)	9.12 s (SD 1.16)

## Data Availability

The original contributions presented in this study are included in the article. Further inquiries can be directed to the corresponding author.

## Abbreviations

The following abbreviations are used in this manuscript:

DOF	Degrees of Freedom
HMI	Human–Machine Interaction
ADAS	Advanced Driver Assistance Systems
ARAS	Advanced Rider Assistance Systems
PTW	Power-Two-Wheeler
HMD	Head-Mounted Display
CAD	Computer-Aided Design
PLC	Programmable Logic Controller
CoG	Center of Gravity
FPS	Frames Per Second
CTU	Czech Technical University
UDP	User Datagram Protocol
HTTP	HyperText Transfer Protocol
GNSS	Global Navigation Satellite System
SD	Standard Deviation
*p* Value	Probability Value

## Appendix A. Experiment Questionnaire

**(1) Before the Experiment**.

Question	Response
Do you own a motorcycle?	
How many years of motorcycle experience do you have?	
Age:	
Gender:	

**(2) General Feedback After the Experiment**.

Question	Motion platform off (static)	Motion platform on (dynamic)
(1) How did you perceive the overall sense of speed?	1 2 3 4 5 6 7 8 9	1 2 3 4 5 6 7 8 9
(2) How did you perceive surface irregularities?	1 2 3 4 5 6 7 8 9	1 2 3 4 5 6 7 8 9
(3) How did you perceive overall braking?	1 2 3 4 5 6 7 8 9	1 2 3 4 5 6 7 8 9
(4) How did you perceive overall acceleration?	1 2 3 4 5 6 7 8 9	1 2 3 4 5 6 7 8 9
(5) How did you perceive visual changes in speed?	1 2 3 4 5 6 7 8 9	1 2 3 4 5 6 7 8 9
(6) How do you rate the motion platform feedback during braking and acceleration?	-	1 2 3 4 5 6 7 8 9
(7) Overall feeling when riding in a straight line?	1 2 3 4 5 6 7 8 9	1 2 3 4 5 6 7 8 9
(8) Was the acceleration proportional to the throttle input?	1 2 3 4 5 6 7 8 9	1 2 3 4 5 6 7 8 9
(9) How did you perceive motion platform feedback during leaning?	-	1 2 3 4 5 6 7 8 9
(10)How did you perceive the leaning in the visualization?	1 2 3 4 5 6 7 8 9	1 2 3 4 5 6 7 8 9
(11) How would you rate the handlebar feedback?	1 2 3 4 5 6 7 8 9	1 2 3 4 5 6 7 8 9
(12) Was the footpeg force feedback adequate?	1 2 3 4 5 6 7 8 9	1 2 3 4 5 6 7 8 9
(13) Overall perception of cornering?	1 2 3 4 5 6 7 8 9	1 2 3 4 5 6 7 8 9
(14) Did you feel motion sickness during the ride?	1 2 3 4 5 6 7 8 9	1 2 3 4 5 6 7 8 9

**(3) Minimal Scenario–SLALOM**.

Question	Motion platform off (static)	Motion platform on (dynamic)
(15) Subjective difficulty?	1 2 3 4 5 6 7 8 9	1 2 3 4 5 6 7 8 9
(16) Sense of control over the motorcycle?	1 2 3 4 5 6 7 8 9	1 2 3 4 5 6 7 8 9
(17) Self-assessment of performance?	1 2 3 4 5 6 7 8 9	1 2 3 4 5 6 7 8 9
(18) Smoothness of the ride?	1 2 3 4 5 6 7 8 9	1 2 3 4 5 6 7 8 9

**(4) Minimal Scenario–LONG CORNER (LEFT AND RIGHT)**.

Question	Motion platform off (static)	Motion platform on (dynamic)
(19) Subjective difficulty?	1 2 3 4 5 6 7 8 9	1 2 3 4 5 6 7 8 9
(20) Sense of control over the motorcycle?	1 2 3 4 5 6 7 8 9	1 2 3 4 5 6 7 8 9
(21) Self-assessment of performance?	1 2 3 4 5 6 7 8 9	1 2 3 4 5 6 7 8 9
(22) Smoothness of the ride?	1 2 3 4 5 6 7 8 9	1 2 3 4 5 6 7 8 9

**(5) Minimal Scenario–EVASIVE MANEUVER**.

Question	Motion platform off (static)	Motion platform on (dynamic)
(23) Subjective difficulty?	1 2 3 4 5 6 7 8 9	1 2 3 4 5 6 7 8 9
(24) Sense of control over the motorcycle?	1 2 3 4 5 6 7 8 9	1 2 3 4 5 6 7 8 9
(25) Self-assessment of performance?	1 2 3 4 5 6 7 8 9	1 2 3 4 5 6 7 8 9
(26) Smoothness of the ride?	1 2 3 4 5 6 7 8 9	1 2 3 4 5 6 7 8 9

**(6) Open Feedback**.

Question	Open response
(27) Do you have any additional comments or suggestions?	
